# Supplementary material for: MOSES: A New Approach to Integrate Interactome Topology and Functional Features for Disease Gene Prediction
Source: Genes (Basel). 2021 Oct 27;12(11):1713. doi: 10.3390/genes12111713 (PMC8624742; doi:10.3390/genes12111713)
Supplement: Supplementary file 1 [file genes-12-01713-s001.zip › genes-1413627-supplementary/genes-1413627supplementary.v1/Figure S1-21.pdf]

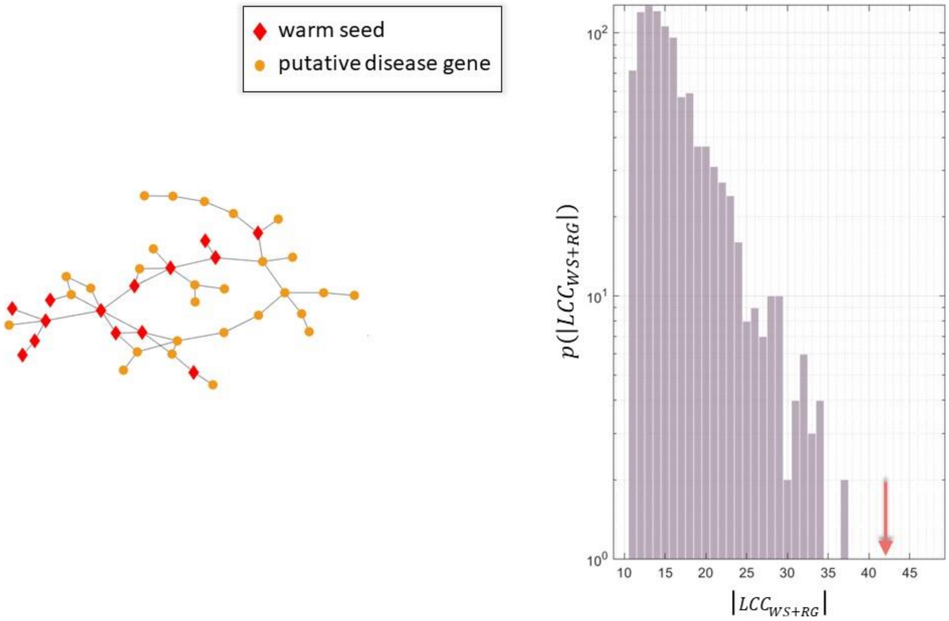

Figure S1. Largest connected component (*LCC*) of the predicted disease module (*warm seeds* and putative disease genes) for *amino acid metabolism inborn errors*. Node shape codes for the type of genes: red diamonds represent the *warm seeds* (14 nodes), while orange dots represent the putative disease genes (28 nodes). On the right, distribution of the size of the 1000 *LCCs* of the random disease modules ($|LCC_{WS+RG}|$) obtained adding to the *warm seeds*, a set of randomly selected genes with cardinality equal to the set of putative genes; the orange arrow indicates the size of the LCC_{WS+PG} shown in the left panel.

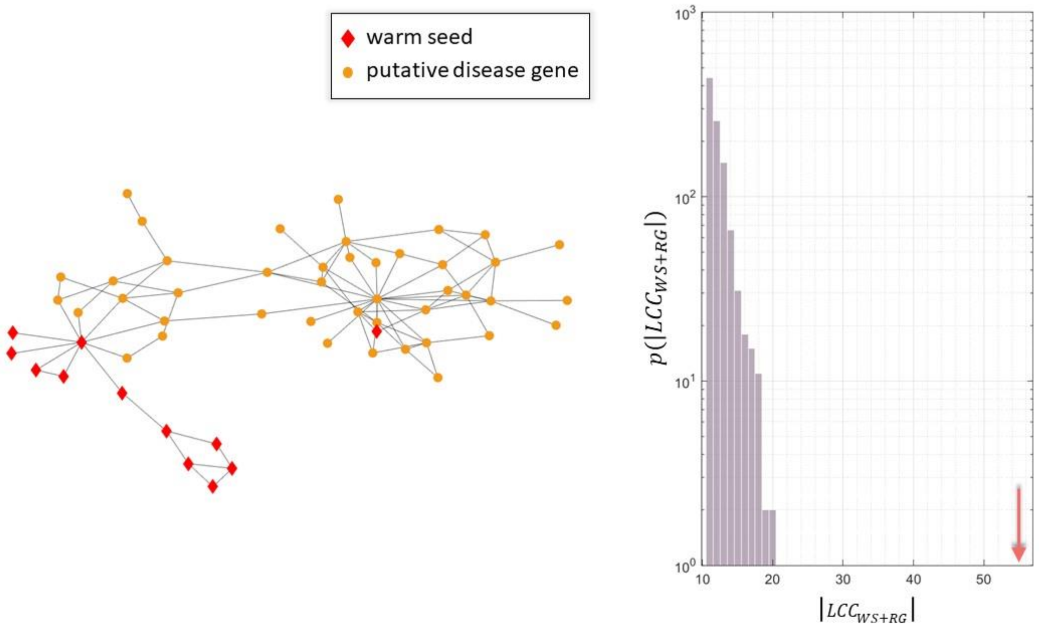

Figure S2. Largest connected component (*LCC*) of the predicted disease module (*warm seeds* and putative disease genes) for *anemia*, *hemolytic*. Node shape codes for the type of genes: red diamonds represent the *warm seeds* (12 nodes), while orange dots represent the putative disease genes (43 nodes). On the right, distribution of the size of the 1000 *LCCs* of the random disease modules ($|LCC_{WS+RG}|$) obtained adding to the *warm seeds*, a set of randomly selected genes with cardinality equal to the set of putative genes; the orange arrow indicates the size of the LCC_{WS+PG} shown in the left panel.

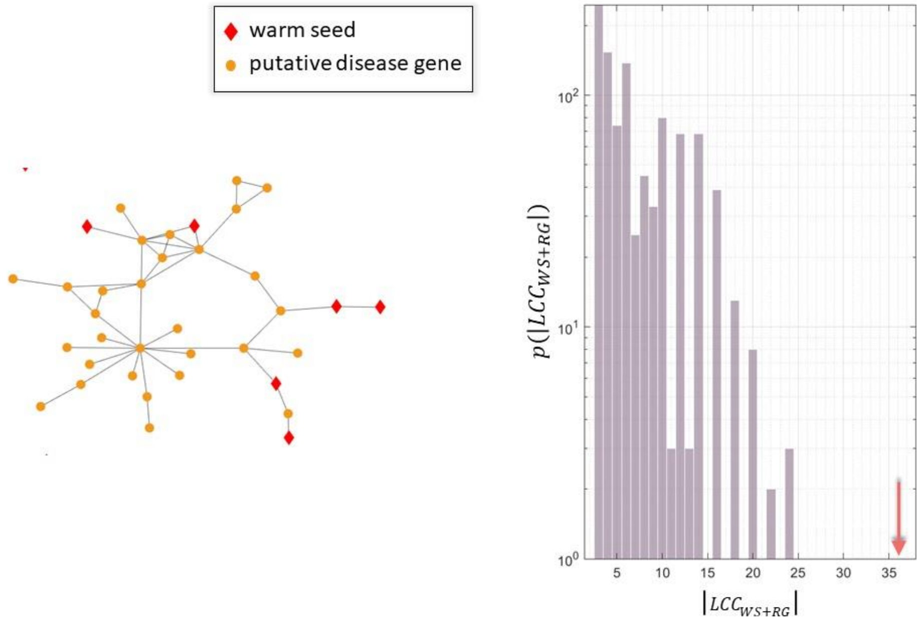

Figure S3. Largest connected component (*LCC*) of the predicted disease module (*warm seeds* and putative disease genes) for *arrhythmias, cardiac*. Node shape codes for the type of genes: red diamonds represent the *warm seeds* (6 nodes), while orange dots represent the putative disease genes (30 nodes). On the right, distribution of the size of the 1000 *LCCs* of the random disease modules ($|LCC_{WS+RG}|$) obtained adding to the *warm seeds*, a set of randomly selected genes with cardinality equal to the set of putative genes; the orange arrow indicates the size of the LCC_{WS+PG} shown in the left panel.

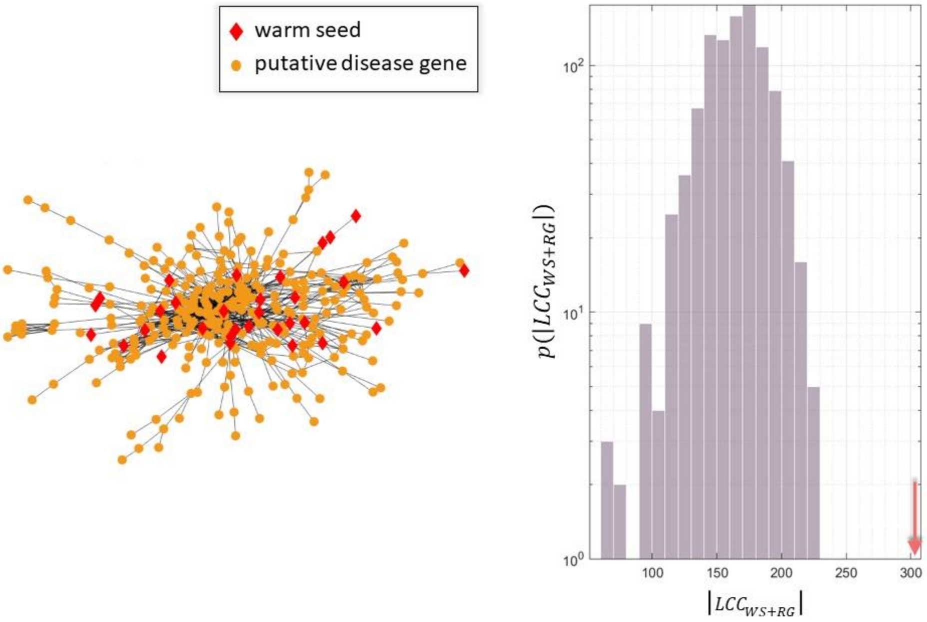

Figure S4. Largest connected component (*LCC*) of the predicted disease module (*warm seeds* and putative disease genes) for *arthritis, rheumatoid*. Node shape codes for the type of genes: red diamonds represent the *warm seeds* (31 nodes), while orange dots represent the putative disease genes (275 nodes). On the right, distribution of the size of the 1000 *LCCs* of the random disease modules ($|LCC_{WS+RG}|$) obtained adding to the *warm seeds*, a set of randomly selected genes with cardinality equal to the set of putative genes; the orange arrow indicates the size of the LCC_{WS+PG} shown in the left panel.

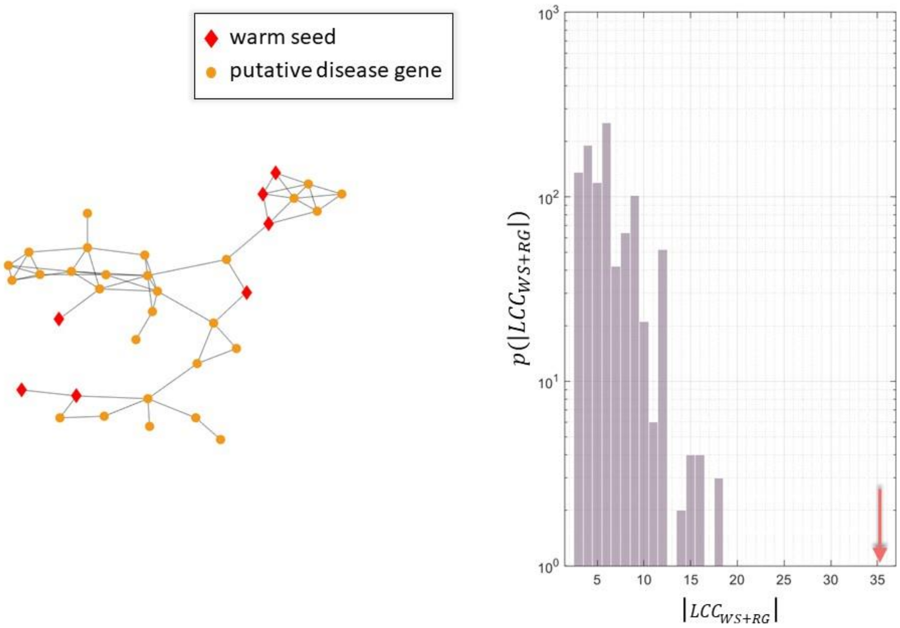

Figure S5. Largest connected component (*LCC*) of the predicted disease module (*warm seeds* and putative disease genes) for *bile duct diseases*. Node shape codes for the type of genes: red diamonds represent the *warm seeds* (7 nodes), while orange dots represent the putative disease genes (28 nodes). On the right, distribution of the size of the 1000 *LCCs* of the random disease modules ($|LCC_{WS+RG}|$) obtained adding to the *warm seeds*, a set of randomly selected genes with cardinality equal to the set of putative genes; the orange arrow indicates the size of the LCC_{WS+PG} shown in the left panel.

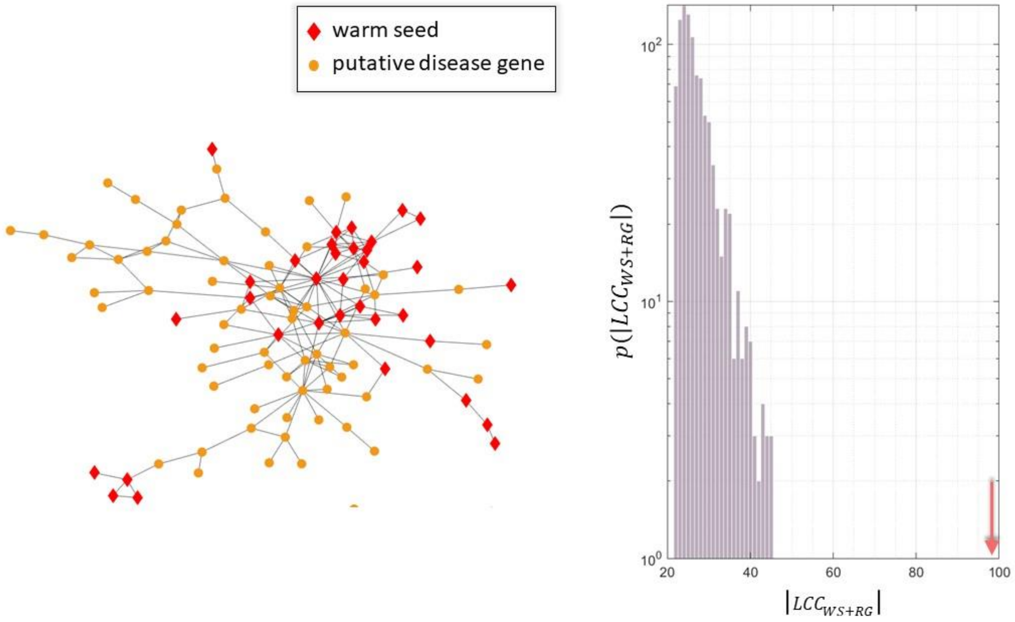

Figure S6. Largest connected component (*LCC*) of the predicted disease module (*warm seeds* and putative disease genes) for *blood coagulation disorders*. Node shape codes for the type of genes: red diamonds represent the *warm seeds* (34 nodes), while orange dots represent the putative disease genes (64 nodes). On the right, distribution of the size of the 1000 *LCCs* of the random disease modules ($|LCC_{WS+RG}|$) obtained adding to the *warm seeds*, a set of randomly selected genes with cardinality equal to the set of putative genes; the orange arrow indicates the size of the LCC_{WS+PG} shown in the left panel.

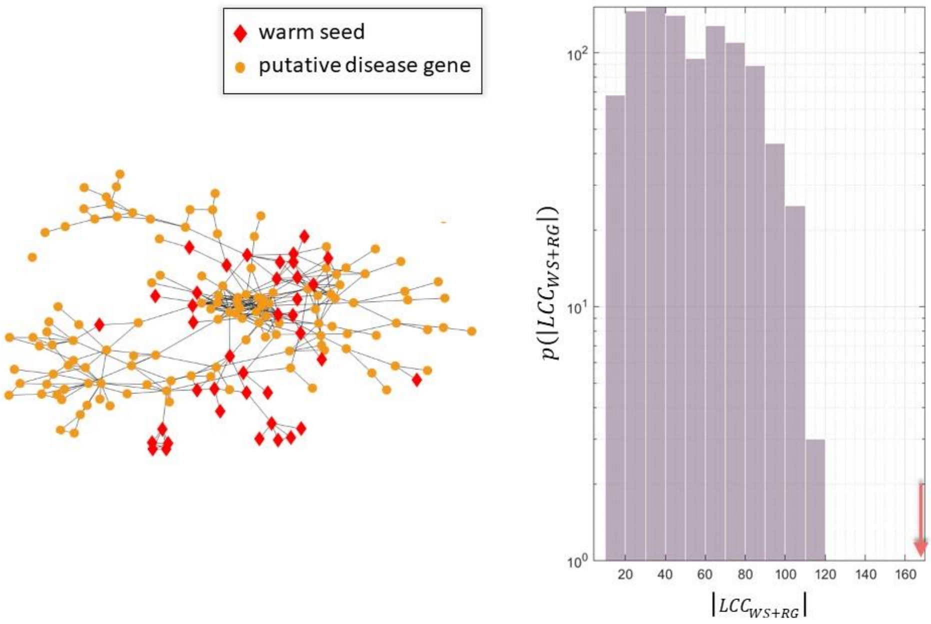

Figure S7. Largest connected component (*LCC*) of the predicted disease module (*warm seeds* and putative disease genes) for *carbohydrate metabolism inborn errors*. Node shape codes for the type of genes: red diamonds represent the *warm seeds* (39 nodes), while orange dots represent the putative disease genes (129 nodes). On the right, distribution of the size of the 1000 *LCCs* of the random disease modules ($|LCC_{WS+RG}|$) obtained adding to the *warm seeds*, a set of randomly selected genes with cardinality equal to the set of putative genes; the orange arrow indicates the size of the LCC_{WS+PG} shown in the left panel.

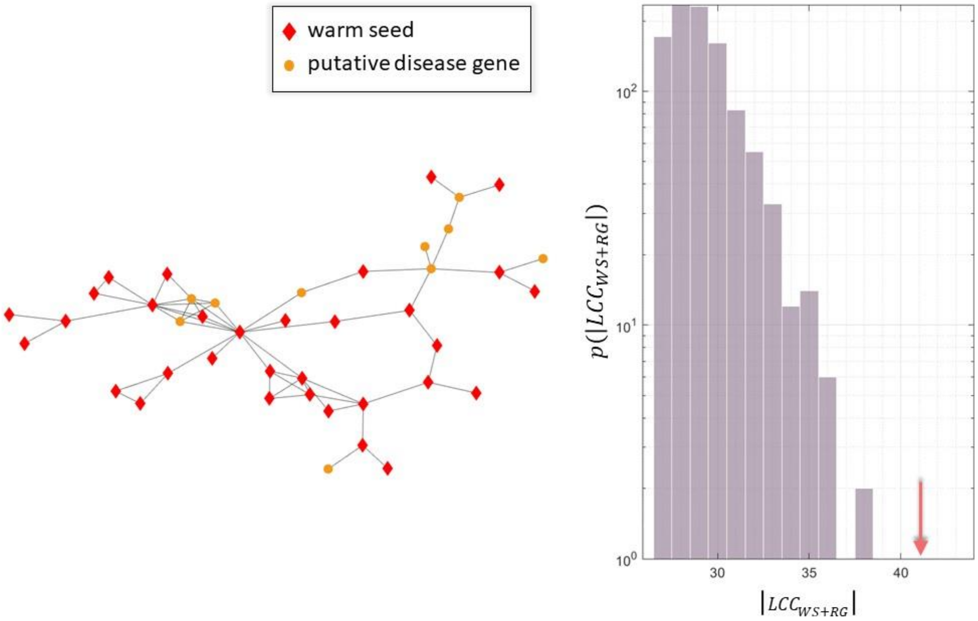

Figure S8. Largest connected component (*LCC*) of the predicted disease module (*warm seeds* and putative disease genes) for *cardiomyopathies*. Node shape codes for the type of genes: red diamonds represent the *warm seeds* (32 nodes), while orange dots represent the putative disease genes (10 nodes). On the right, distribution of the size of the 1000 *LCCs* of the random disease modules ($|LCC_{WS+RG}|$) obtained adding to the *warm seeds*, a set of randomly selected genes with cardinality equal to the set of putative genes; the orange arrow indicates the size of the LCC_{WS+PG} shown in the left panel.

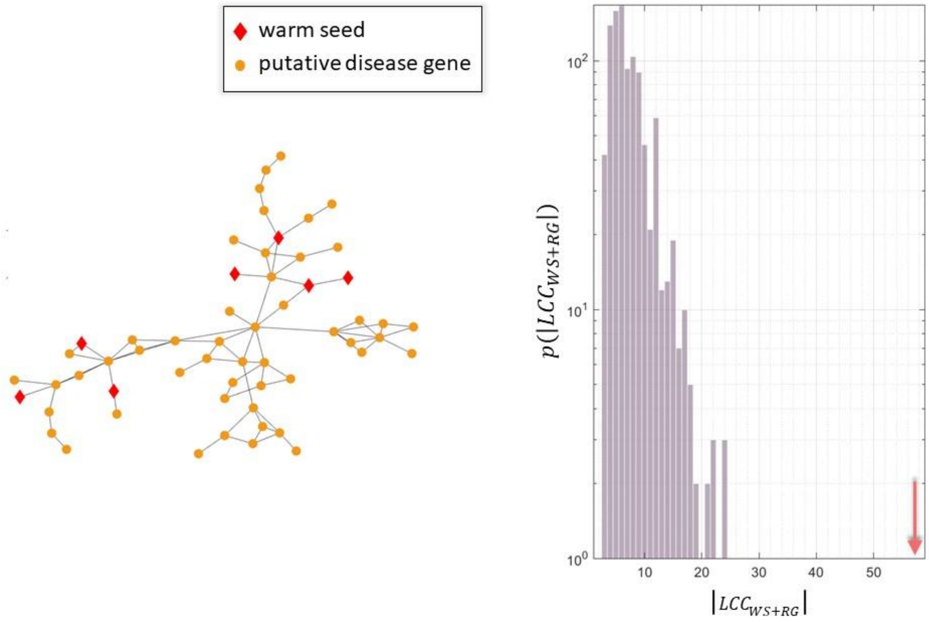

Figure S9. Largest connected component (*LCC*) of the predicted disease module (*warm seeds* and putative disease genes) for *celiac disease*. Node shape codes for the type of genes: red diamonds represent the *warm seeds* (7 nodes), while orange dots represent the putative disease genes (50 nodes). On the right, distribution of the size of the 1000 *LCCs* of the random disease modules ($|LCC_{WS+RG}|$) obtained adding to the *warm seeds*, a set of randomly selected genes with cardinality equal to the set of putative genes; the orange arrow indicates the size of the LCC_{WS+PG} shown in the left panel.

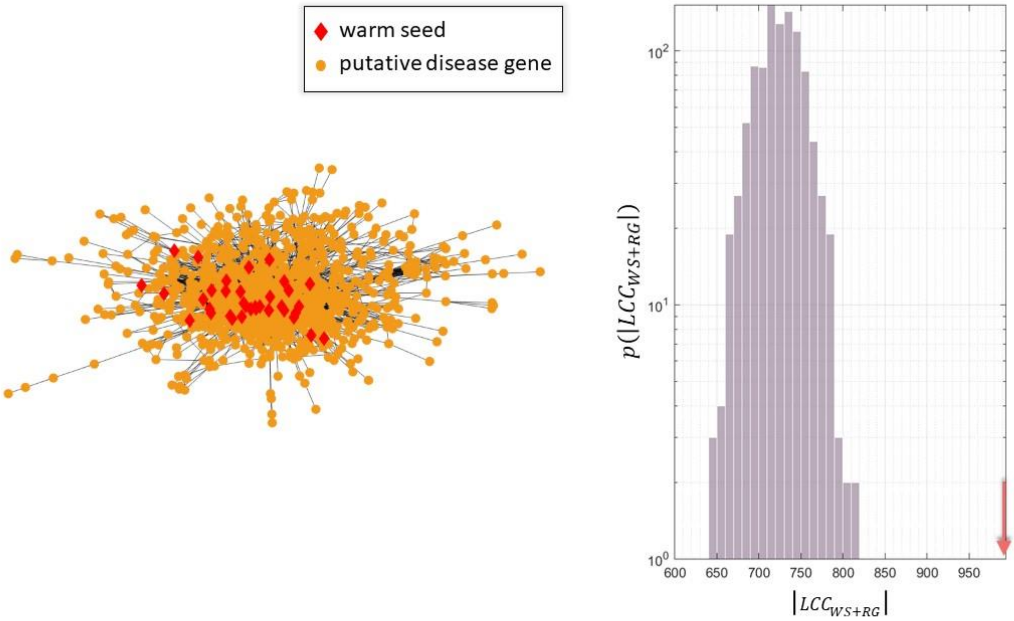

Figure S10. Largest connected component (*LCC*) of the predicted disease module (*warm seeds* and putative disease genes) for *colorectal neoplasms*. Node shape codes for the type of genes: red diamonds represent the *warm seeds* (35 nodes), while orange dots represent the putative disease genes (957 nodes). On the right, distribution of the size of the 1000 *LCCs* of the random disease modules ($|LCC_{WS+RG}|$) obtained adding to the *warm seeds*, a set of randomly selected genes with cardinality equal to the set of putative genes; the orange arrow indicates the size of the LCC_{WS+PG} shown in the left panel.

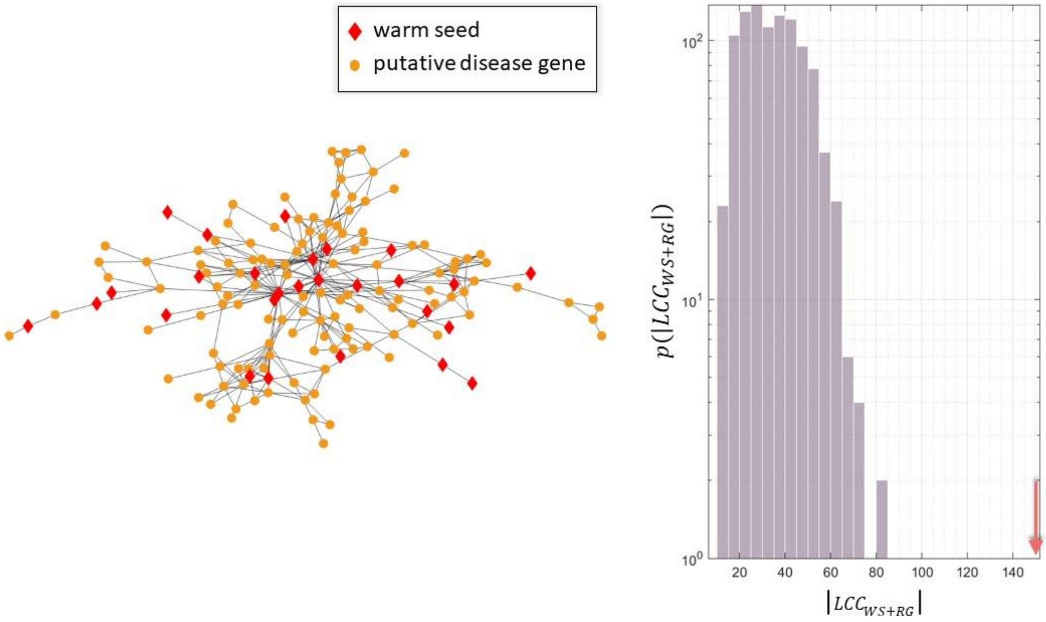

Figure S11. Largest connected component (*LCC*) of the predicted disease module (*warm seeds* and putative disease genes) for *crohn disease*. Node shape codes for the type of genes: red diamonds represent the *warm seeds* (27 nodes), while orange dots represent the putative disease genes (123 nodes). On the right, distribution of the size of the 1000 *LCCs* of the random disease modules ($|LCC_{WS+RG}|$) obtained adding to the *warm seeds*, a set of randomly selected genes with cardinality equal to the set of putative genes; the orange arrow indicates the size of the LCC_{WS+PG} shown in the left panel.

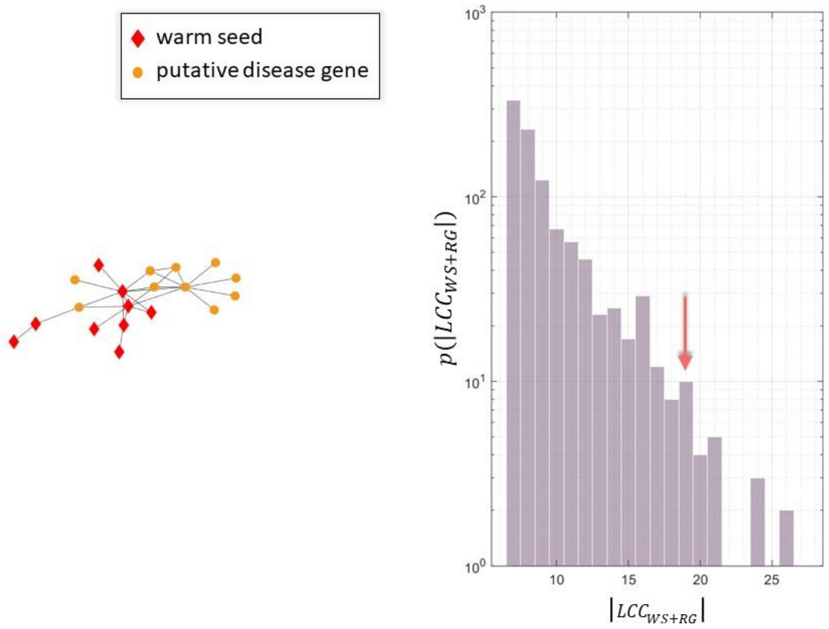

Figure S12. Largest connected component (*LCC*) of the predicted disease module (*warm seeds* and putative disease genes) for *diabetes mellitus type 2*. Node shape codes for the type of genes: red diamonds represent the *warm seeds* (9 nodes), while orange dots represent the putative disease genes (10 nodes). On the right, distribution of the size of the 1000 *LCCs* of the random disease modules ($|LCC_{WS+RG}|$) obtained adding to the *warm seeds*, a set of randomly selected genes with cardinality equal to the set of putative genes; the orange arrow indicates the size of the LCC_{WS+PG} shown in the left panel.

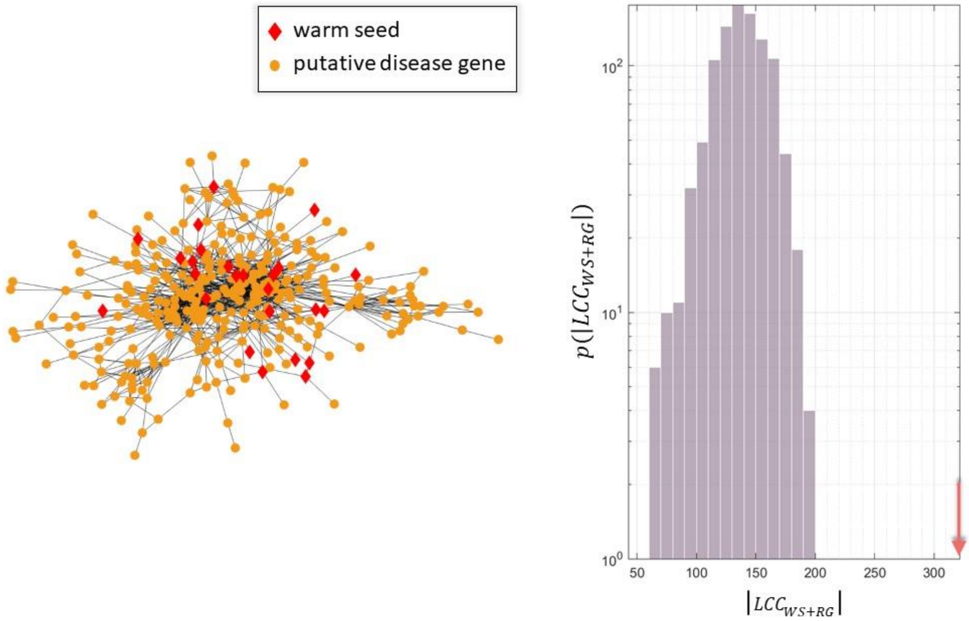

Figure S13. Largest connected component (*LCC*) of the predicted disease module (*warm seeds* and putative disease genes) for *head and neck neoplasms*. Node shape codes for the type of genes: red diamonds represent the *warm seeds* (25 nodes), while orange dots represent the putative disease genes (295 nodes). On the right, distribution of the size of the 1000 *LCCs* of the random disease modules ($|LCC_{WS+RG}|$) obtained adding to the *warm seeds*, a set of randomly selected genes with cardinality equal to the set of putative genes; the orange arrow indicates the size of the LCC_{WS+PG} shown in the left panel.

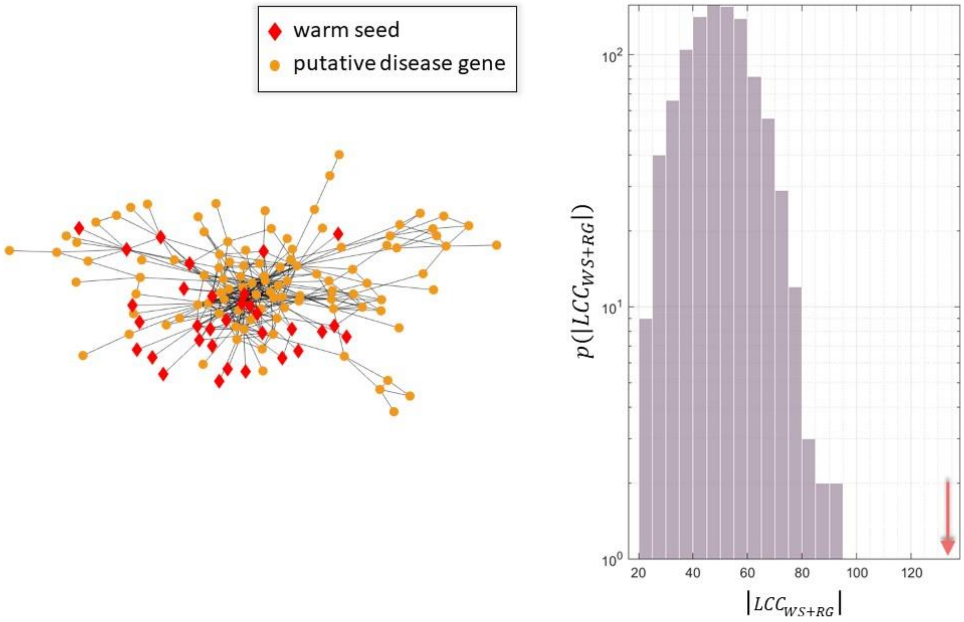

Figure S14. Largest connected component (*LCC*) of the predicted disease module (*warm seeds* and putative disease genes) for *leukemia, myeloid*. Node shape codes for the type of genes: red diamonds represent the *warm seeds* (32 nodes), while orange dots represent the putative disease genes (104 nodes). On the right, distribution of the size of the 1000 *LCCs* of the random disease modules ($|LCC_{WS+RG}|$) obtained adding to the *warm seeds*, a set of randomly selected genes with cardinality equal to the set of putative genes; the orange arrow indicates the size of the LCC_{WS+PG} shown in the left panel.

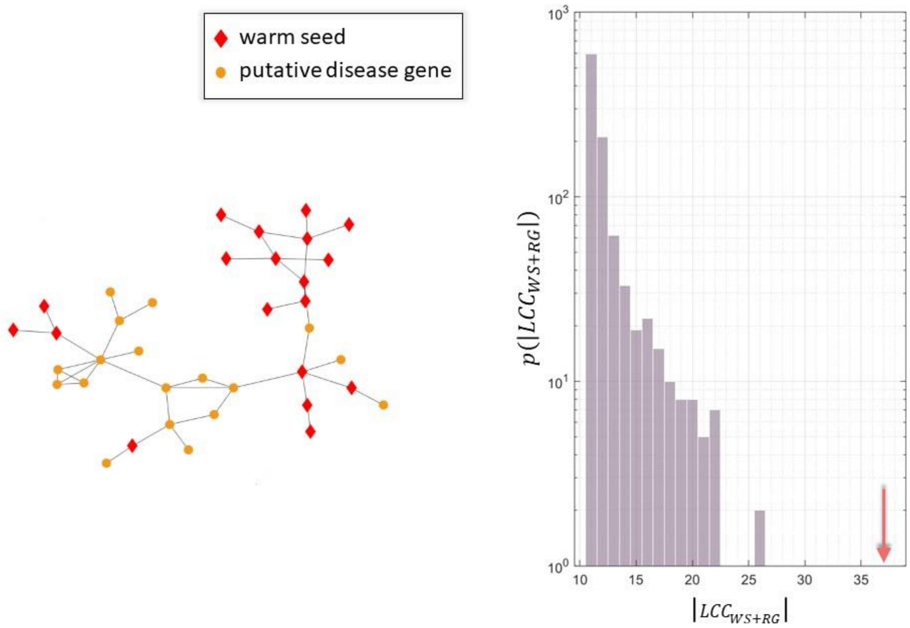

Figure S15. Largest connected component (*LCC*) of the predicted disease module (*warm seeds* and putative disease genes) for *lipid metabolism disorders*. Node shape codes for the type of genes: red diamonds represent the *warm seeds* (19 nodes), while orange dots represent the putative disease genes (18 nodes). On the right, distribution of the size of the 1000 *LCCs* of the random disease modules ($|LCC_{WS+RG}|$) obtained adding to the *warm seeds*, a set of randomly selected genes with cardinality equal to the set of putative genes; the orange arrow indicates the size of the LCC_{WS+PG} shown in the left panel.

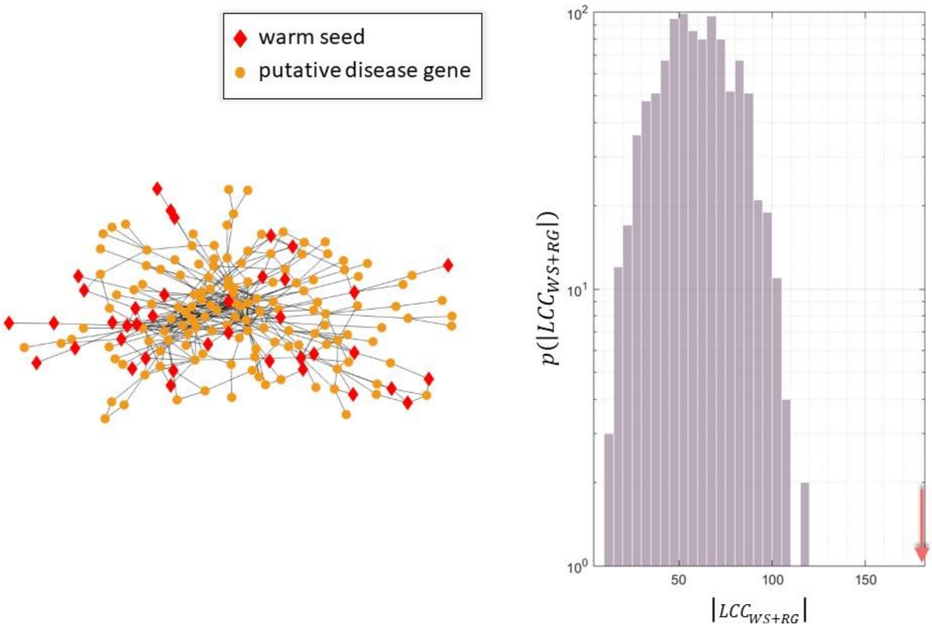

Figure S16. Largest connected component (*LCC*) of the predicted disease module (*warm seeds* and putative disease genes) for *lupus erythematosus*. Node shape codes for the type of genes: red diamonds represent the *warm seeds* (39 nodes), while orange dots represent the putative disease genes (141 nodes). On the right, distribution of the size of the 1000 *LCCs* of the random disease modules ($|LCC_{WS+RG}|$) obtained adding to the *warm seeds*, a set of randomly selected genes with cardinality equal to the set of putative genes; the orange arrow indicates the size of the LCC_{WS+PG} shown in the left panel.

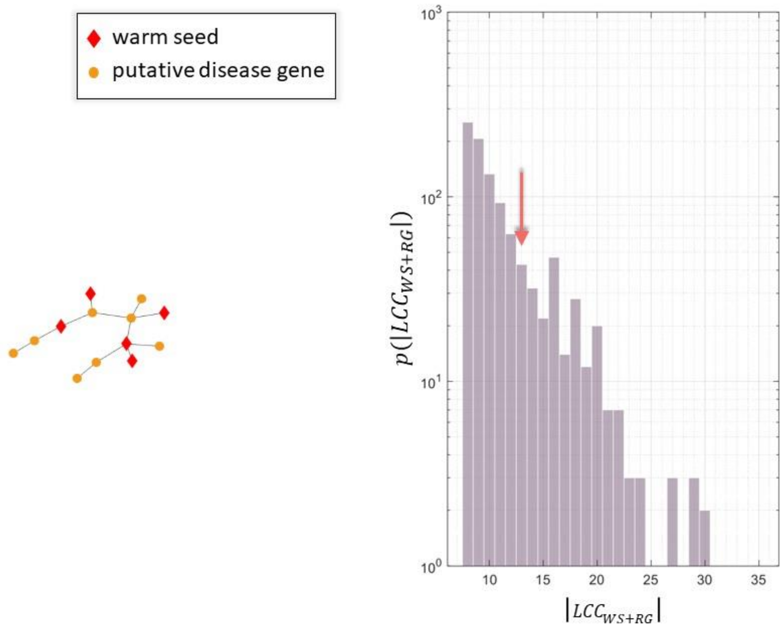

Figure S17. Largest connected component (*LCC*) of the predicted disease module (*warm seeds* and putative disease genes) for *lysosomal storage diseases*. Node shape codes for the type of genes: red diamonds represent the *warm seeds* (5 nodes), while orange dots represent the putative disease genes (8 nodes). On the right, distribution of the size of the 1000 *LCCs* of the random disease modules ($|LCC_{WS+RG}|$) obtained adding to the *warm seeds*, a set of randomly selected genes with cardinality equal to the set of putative genes; the orange arrow indicates the size of the LCC_{WS+PG} shown in the left panel.

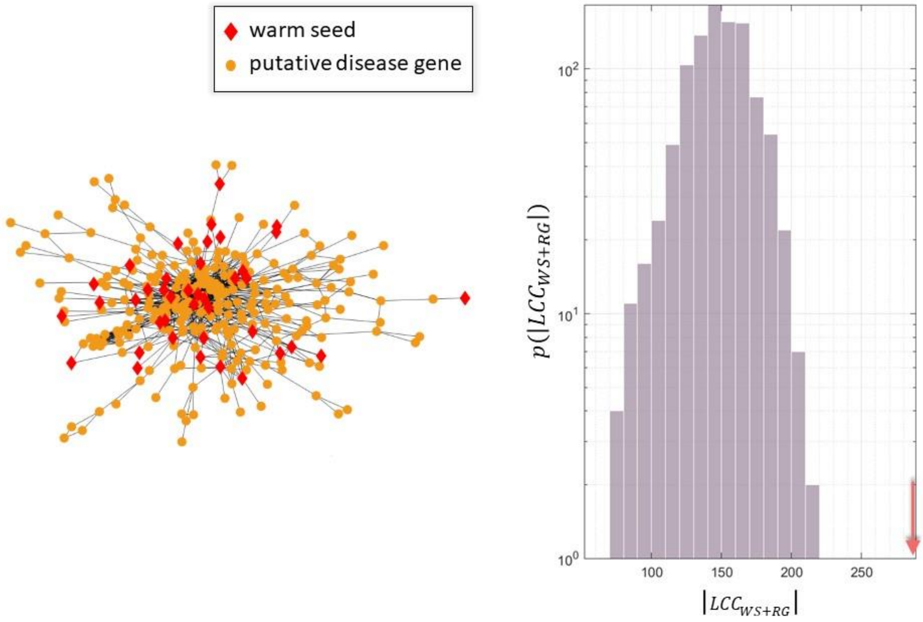

Figure S18. Largest connected component (*LCC*) of the predicted disease module (*warm seeds* and putative disease genes) for *multiple sclerosis*. Node shape codes for the type of genes: red diamonds represent the *warm seeds* (40 nodes), while orange dots represent the putative disease genes (247 nodes). On the right, distribution of the size of the 1000 *LCCs* of the random disease modules ($|LCC_{WS+RG}|$) obtained adding to the *warm seeds*, a set of randomly selected genes with cardinality equal to the set of putative genes; the orange arrow indicates the size of the LCC_{WS+PG} shown in the left panel.

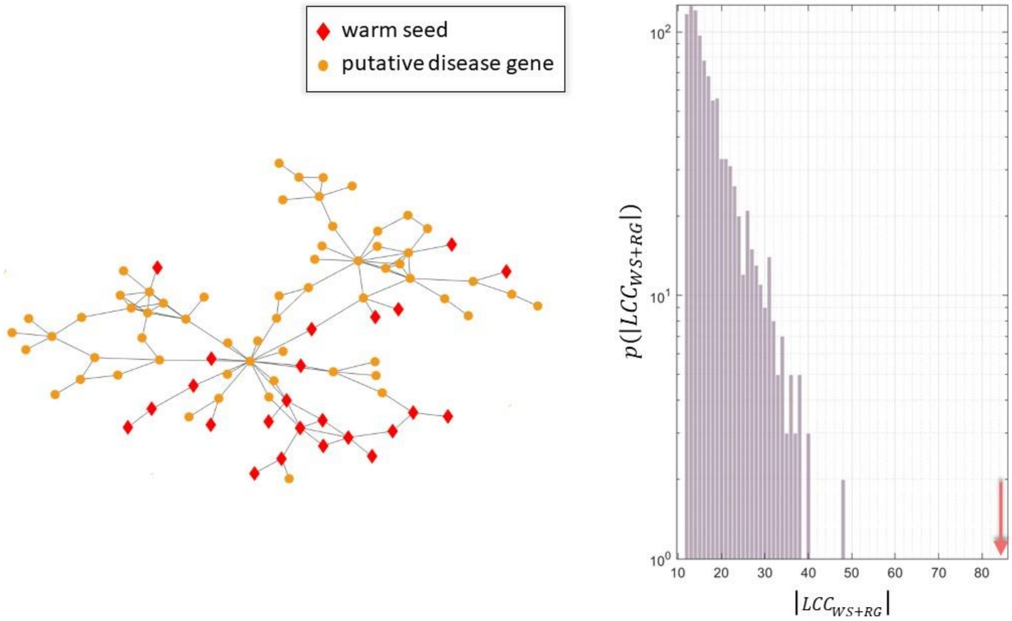

Figure S19. Largest connected component (*LCC*) of the predicted disease module (*warm seeds* and putative disease genes) for *muscular dystrophies*. Node shape codes for the type of genes: red diamonds represent the *warm seeds* (24 nodes), while orange dots represent the putative disease genes (60 nodes). On the right, distribution of the size of the 1000 *LCCs* of the random disease modules ($|LCC_{WS+RG}|$) obtained adding to the *warm seeds*, a set of randomly selected genes with cardinality equal to the set of putative genes; the orange arrow indicates the size of the LCC_{WS+PG} shown in the left panel.

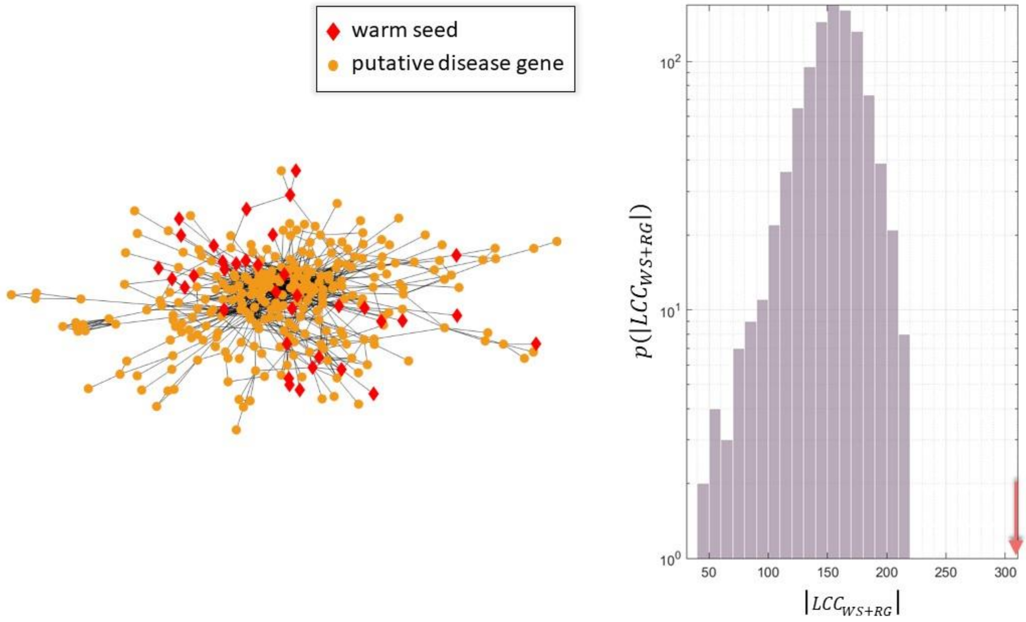

Figure S20. Largest connected component (*LCC*) of the predicted disease module (*warm seeds* and putative disease genes) for *psoriasis*. Node shape codes for the type of genes: red diamonds represent the *warm seeds* (36 nodes), while orange dots represent the putative disease genes (273 nodes). On the right, distribution of the size of the 1000 *LCCs* of the random disease modules ($|LCC_{WS+RG}|$) obtained adding to the *warm seeds*, a set of randomly selected genes with cardinality equal to the set of putative genes; the orange arrow indicates the size of the LCC_{WS+PG} shown in the left panel.

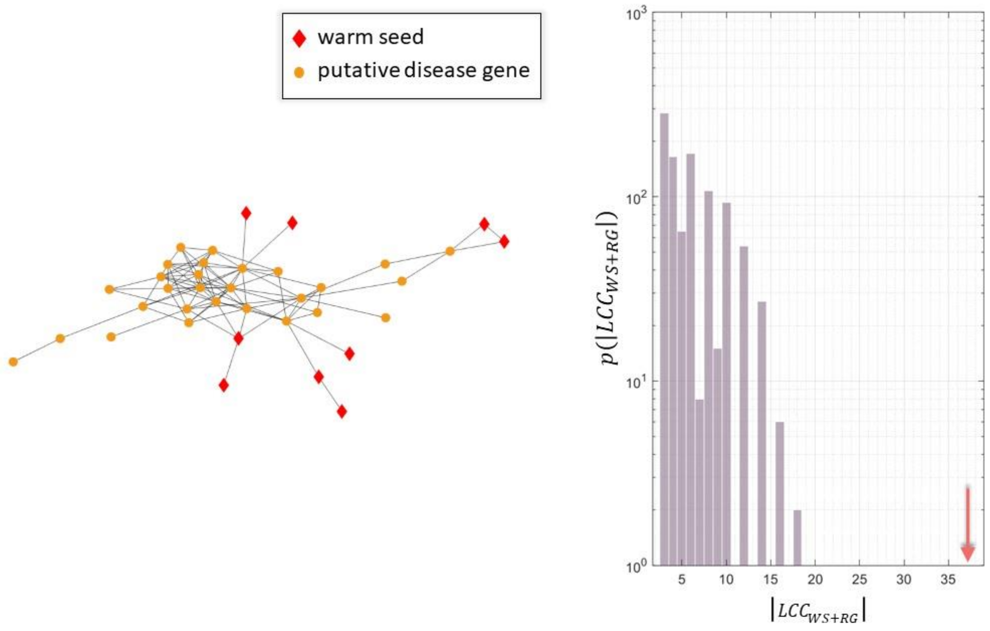

Figure S21. Largest connected component (*LCC*) of the predicted disease module (*warm seeds* and putative disease genes) for *spinocerebellar degenerations*. Node shape codes for the type of genes: red diamonds represent the *warm seeds* (9 nodes), while orange dots represent the putative disease genes (28 nodes). On the right, distribution of the size of the 1000 *LCCs* of the random disease modules ($|LCC_{WS+RG}|$) obtained adding to the *warm seeds*, a set of randomly selected genes with cardinality equal to the set of putative genes; the orange arrow indicates the size of the LCC_{WS+PG} shown in the left panel.
